# Iron and Folic Acid Supplementation Affects Mineral Status in Female Rats with a Deficiency of These Micronutrients

**DOI:** 10.1007/s12011-020-02460-w

**Published:** 2020-10-28

**Authors:** Joanna Suliburska, Katarzyna Skrypnik, Agata Chmurzyńska

**Affiliations:** Department of Human Nutrition and Dietetics, Poznan University of Life Science, ul. Wojska Polskiego 31, 60-624 Poznań, Poland

**Keywords:** Iron, Folic acid, Minerals, Rat

## Abstract

Supplementation with iron and folic acid is widely recommended in women of childbearing age and during pregnancy; however, the effect of such supplementation on mineral status is not well-known. The aim of this study was to determine the effects of oral iron and folic acid, administered together and separately, on copper, zinc, calcium, and magnesium concentrations in the tissues of rats with a deficiency of both these micronutrients. The experiment was performed on 8-week-old female Wistar rats. In the first stage of the experiment, the animals were randomly assigned to a control group of rats fed the standard diet (AIN-93 M), and to a study group of rats fed a diet deficient in iron and folate. The study group was then randomly divided to four groups: group D was fed a deficit diet, group FE was fed a deficit diet with iron gluconate, the FOL group was fed a deficit diet with folate acid, and the FEFOL group was fed a deficit diet with iron gluconate and folate acid. After 2, 10, and 21 days of the intervention, ten animals from each group were killed. Mineral concentrations were assayed in the liver, spleen, pancreas, heart, and kidneys using atomic absorption spectrometry. Statistical analysis was performed using Statistica 12.0 with the ANOVA test (*p* < 0.05). It was found that separate supplementation with iron and folic acid significantly decreased copper concentrations in tissues. The deficit in iron and folic acid decreased, and their simultaneous supplementation increased calcium content in the organs. Separate and simultaneous supplementation decreased magnesium status in deficient rats. In conclusion, iron and folic acid, supplemented separately or simultaneously, affect the copper, calcium, and magnesium level in tissues.

## Introduction

Iron and folic acid deficiency is common among women of childbearing age, and also in pregnant women. The World Health Organization (WHO) has estimated that more than 30% of women of reproductive age worldwide are anemic, and that at least half of these have iron deficiency anemia [1]. The prevalence of anemia in pregnant women is also high (40% or more). The main reason for this health problem is chronic iron depletion during the menstrual cycle and accompanying iron and folic acid malnutrition. The inadequate intake of these micronutrients among young women has been well documented in many developed countries. Low levels of iron and folate have been also found in the daily food rations of pregnant women, which is associated with an increased risk of anemia for the mother, and of neural tube defects for the fetus [1, 2]. Iron and folic acid supplementation is therefore recommended as a public health intervention in menstruating and pregnant women, in order to improve their hemoglobin concentrations and iron and folic acid status, and thus reducing the risk of anemia [3]. Although iron and folate deficiency is a well-known global health problem, it is worth highlighting that micronutrient deficiencies often coexist, and that deficiency of copper, zinc, calcium, and magnesium can lead to anemia, osteoporosis, neural defects, and poor pregnancy outcomes, such as preeclampsia or low birth weight [4, 5]. Mineral status may have an effect on iron and folate supplementation effectiveness. On the other hand, iron-folic acid supplementation may affect the status of other micronutrients in the body through interaction processes. In our previous paper, we described the interaction between folic acid and iron, which resulted in decreased iron status in female rats deficient in these micronutrients undergoing moderate and long-term supplementation [6]. We found that dietary folic acid and iron deficiency, and subsequent supplementation of these micronutrients, affected transcription levels, but not protein levels, of folic acid and iron transporters in the duodenum and liver [7, 8]. The interactions between calcium, zinc, copper, and iron have been widely documented, and occur mainly in the stages of absorption, transport, and metabolism of these minerals [9]. Copper and iron metabolism are strongly related, especially through the activity of ceruloplasmin and hephaestin. Ceruloplasmin plays a role in iron redistribution from the liver and other tissues. Hephaestin is a ceruloplasmin homolog and plays a role in the uptake of iron from the diet. Current results suggest that the deletion of hephaestin and ceruloplasmin leads to systemic iron deficiency and iron overload in local tissues, which disrupts iron homeostasis [10, 11]. The effect of elements (such as calcium and zinc) on iron homeostasis may be mediated through its influence on iron absorption, circulating transporters, and regulation of hepcidin production [12, 13]. Moreover, excess zinc in the diet is known to induce copper deficiency, leading to a reduction in iron bioavailability, eventually resulting in anemia. Several transporters involved in iron, copper, and zinc metabolism may be associated with the absorption of other elements [14].

To determine the effects of iron and folic acid supplementation on mineral status, it is necessary to demonstrate the effectiveness and safety of this supplementation, which is widely recommended for young women. Although iron and folic acid supplements are commonly used, little is known about their effects on mineral status in the body. The aim of this study was thus to investigate the impact of separate and simultaneous iron and folic acid supplementation on copper, zinc, calcium, and magnesium concentration in the tissues of rats with a deficiency of both these micronutrients.

## Material and Methods

### Animals

The study protocol was approved by the local bioethics committee (approval no. 59/2016).

One hundred fifty 8-week-old female Wistar rats were obtained from AnimaLab (Germany) directly before the experiment. The mean weight of the rats at baseline was 182.9 g. Details of the animal breeding conditions were described in our previous paper [15].

### Experimental Design

The animals were adapted to laboratory conditions over the first 5 days. In the first stage of the experiment, the animals were randomly assigned to two groups: deficient (*n* = 120 rats) and control (*n* = 30 rats). The animals were fed a semisynthetic diet based on the AIN-93 M diet (Reeves 1997). Rats in the deficient group were fed an iron- and folate-deficit diet, while those in the control group were fed the standard diet. All rats were provided ad libitum diet and distilled water for 28 days. After that time, in the second stage, the deficient group (D) was randomly divided into four groups of 30 rats each. Group D continued to intake the deficit diet, group FE was fed a standard diet with iron gluconate (150 mg Fe/kg diet), group FOL was fed a standard diet with folate acid (6 mg/kg diet), and group FEFOL was fed a standard diet with iron gluconate (150 mg Fe/kg diet) and folate acid (6 mg/kg diet). The control group (C) was fed the standard diet. The intake of the diets was monitored daily.

After 2 days, and then after 10 days and 21 days of the experiment, ten animals of each group were anesthetized and killed by cardiac puncture, after 12 h of fasting.

The liver, kidney, heart, spleen, and pancreas were dissected, weighted, and stored at − 80 °C. Further details of the experimental design are presented in our previous paper [15].

### Determination of Minerals

The copper, zinc, calcium, and magnesium content of the diet and tissues was determined following digestion in 65% (*w*/*w*) spectra pure HNO_3_ (Merck) in a Microwave Digestion System (Speedwave XPERT microwave digestion system, Berghof, Eningen, Germany). Thereafter, the concentration of minerals was measured using flame atomic absorption spectrometry (Atomic Absorption Spectrophotometer ZA3000, Hitachi, Tokyo, Japan). The accuracy of the method was verified using certified reference materials: Brown Bread BCR191 for the diet samples, Sigma-Aldrich and bovine liver-trace elements, NIST-1577C, CERT for tissue samples. The accuracies proved to be 103–105% for Cu, 90–95% for Zn, 91–93% for Ca, and 98–101% for Mg.

### Statistical Analysis

Detailed statistical analysis was performed using Statistica for Windows 12.0. (StatSoft, Poland). The data were expressed as arithmetic means with standard errors. Comparison between groups was carried out using one-way ANOVA analysis of variance with Turkey’s post hoc test. A Pearson correlation test was performed to calculate correlation coefficients. The significance was set to the *p* < 0.05 level.

## Results

The iron status results have been presented in our previous paper [15], where we found that folic acid supplementation more significantly decreased iron concentrations in the pancreas and spleen than in the deficient group after 10 and 21 days of supplementation. Moreover, the combination of iron with folic acid markedly decreased the iron content of the liver and spleen, in comparison with iron alone, after 10 and 21 days of the experiment [15].

The mineral concentrations measured in the diet samples are shown in Table 1. The daily intake of diet and minerals was comparable between groups (Table 2). The content of copper, zinc, calcium, and magnesium in the tissues is shown in Tables 3, 4, 5, 6.

**Table 1:** Content of minerals in the diets (mg/kg diet)

Group	Copper	Zinc	Calcium	Magnesium
C	7.87 ± 0.28	37.74 ± 2.68	5425.10 ± 39.60	574.15 ± 4.98
D	7.90 ± 0.42	37.21 ± 0.26	5423.76 ± 23.96	575.52 ± 8.64
FE	7.88 ± 0.23	37.73 ± 0.48	5423.62 ± 67.49	574.86 ± 4.03
FOL	7.85 ± 0.54	37.81 ± 0.98	5405.48 ± 67.48	574.01 ± 2.34
FEFOL	7.89 ± 0.34	37.73 ± 1.43	5431.96 ± 37.70	574.93 ± 7.06

*C* control diet, *D* diet deficient in iron and folic acid, *FE* diet deficient in folic acid and supplemented with iron, *FOL* diet deficient in iron and supplemented with folic acid, *FEFOL* diet supplemented with iron and folic acid

**Table 2:** Daily intake of the diet (g) and minerals (mg) in rats

Group	Diet	Copper	Zinc	Calcium	Magnesium
2 days					
C	19.72 ± 0.97	0.16 ± 0.01	0.74 ± 0.05	106.98 ± 0.78	11.32 ± 0.10
D	20.30 ± 0.99	0.16 ± 0.01	0.76 ± 0.01	110.10 ± 0.48	11.68 ± 0.17
FE	19.03 ± 0.60	0.15 ± 0.00	0.72 ± 0.01	103.21 ± 0.31	10.94 ± 0.08
FOL	19.59 ± 0.69	0.15 ± 0.01	0.74 ± 0.02	105.89 ± 1.32	11.24 ± 0.05
FEFOL	20.53 ± 0.72	0.16 ± 0.01	0.77 ± 0.03	111.51 ± 0.77	11.80 ± 0.14
10 days					
C	20.31 ± 0.86	0.16 ± 0.01	0.77 ± 0.05	110.18 ± 0.80	11.66 ± 0.10
D	20.75 ± 0.81	0.16 ± 0.01	0.76 ± 0.01	110.16 ± 0.49	11.69 ± 0.18
FE	19.54 ± 0.83	0.15 ± 0.00	0.74 ± 0.01	105.98 ± 0.32	11.23 ± 0.08
FOL	19.92 ± 0.33	0.15 ± 0.01	0.75 ± 0.02	107.68 ± 1.34	11.43 ± 0.05
FEFOL	20.28 ± 0.76	0.16 ± 0.01	0.77 ± 0.03	110.16 ± 0.76	11.66 ± 0.14
21 days					
C	19.91 ± 0.90	0.16 ± 0.01	0.75 ± 0.05	108.01 ± 0.79	11.43 ± 0.10
D	20.18 ± 0.73	0.16 ± 0.01	0.75 ± 0.01	109.45 ± 0.48	11.61 ± 0.17
FE	19.73 ± 0.61	0.16 ± 0.01	0.74 ± 0.01	107.01 ± 0.32	11.34 ± 0.08
FOL	18.96 ± 1.01	0.15 ± 0.01	0.72 ± 0.02	102.49 ± 1.28	10.88 ± 0.04
FEFOL	20.98 ± 0.76	0.17 ± 0.01	0.79 ± 0.03	113.96 ± 0.79	12.06 ± 0.15

ANOVA and Tukey’s test (*p* > 0.05; non-significant differences within the columns)

*C* control diet, *D* diet deficient in iron and folic acid, *FE* diet deficient in folic acid and supplemented with iron, *FOL* diet deficient in iron and supplemented with folic acid, *FEFOL* diet supplemented with iron and folic acid

**Table 3:** Concentration of copper (μg/g d.w.) in tissues in rats

Group	Liver	Spleen	Pancreas	Kidney	Heart
2 days					
C	19.71 ± 1.44	8.51 ± 0.97	4.33 ± 0.57	27.03 ± 7.09	25.02 ± 1.50 ^b^
D	19.21 ± 2.47	8.84 ± 0.92	4.46 ± 0.45	27.60 ± 5.00	22.51 ± 0.66 ^ab^
FE	19.78 ± 4.31	7.97 ± 1.31	4.35 ± 0.48	24.59 ± 5.10	22.64 ± 2.05 ^ab^
FOL	17.53 ± 2.12	9.53 ± 2.12	4.79 ± 0.57	27.18 ± 4.32	21.34 ± 1.65 ^ab^
FEFOL	20.54 ± 1.55	10.09 ± 1.25	4.51 ± 1.23	23.06 ± 3.81	19.76 ± 6.71 ^a^
10 days					
C	21.99 ± 3.01 ^c^	8.52 ± 1.09	5.11 ± 0.68 ^b^	25.53 ± 4.49 ^b^	25.77 ± 4.11 ^b^
D	20.09 ± 3.59 ^b^	9.08 ± 1.56	4.67 ± 0.52 ^ab^	22.60 ± 3.36 ^ab^	21.59 ± 0.87 ^a^
FE	16.73 ± 0.66 ^ab^	7.93 ± 1.34	4.06 ± 0.54 ^a^	19.42 ± 4.61 ^a^	22.04 ± 1.37 ^a^
FOL	15.25 ± 2.27 ^a^	8.69 ± 1.46	4.76 ± 0.57 ^ab^	25.96 ± 3.10 ^b^	20.40 ± 1.70 ^a^
FEFOL	22.42 ± 2.99 ^c^	8.31 ± 1.41	5.14 ± 1.28 ^b^	20.86 ± 4.19 ^ab^	22.59 ± 1.11 ^a^
21 days					
C	20.72 ± 2.29 ^bc^	8.98 ± 0.87	5.12 ± 0.53 ^b^	24.42 ± 5.76 ^b^	24.69 ± 2.17 ^c^
D	20.84 ± 4.24 ^bc^	8.62 ± 1.14	4.74 ± 0.66 ^ab^	23.86 ± 3.53 ^ab^	22.13 ± 0.93 ^ab^
FE	16.12 ± 1.58 ^a^	7.81 ± 1.79	4.26 ± 0.60 ^a^	18.40 ± 2.44 ^a^	21.31 ± 1.16 ^a^
FOL	16.94 ± 3.23 ^ab^	9.06 ± 0.98	4.90 ± 0.63 ^ab^	27.73 ± 5.29 ^c^	20.67 ± 0.98 ^a^
FEFOL	22.34 ± 2.06 ^c^	8.46 ± 1.26	4.87 ± 0.62 ^ab^	20.77 ± 4.34 ^ab^	23.37 ± 0.79 ^bc^

*C* control diet, *D* diet deficient in iron and folic acid, *FE* diet deficient in folic acid and supplemented with iron, *FOL* diet deficient in iron and supplemented with folic acid, *FEFOL* diet supplemented with iron and folic acid, *d.w*. dry weight

^a, b, c^Significant differences (*p* < 0.05) between groups (within the columns); ANOVA and Tukey’s test

**Table 4:** Concentration of zinc (μg/g d.w.) in tissues in rats

Group	Liver	Spleen	Pancreas	Kidney	Heart
2 days					
C	94.52 ± 4.68 ^ab^	95.32 ± 6.97	87.86 ± 10.83	113.02 ± 11.41	83.11 ± 7.75
D	90.89 ± 11.07 ^a^	99.64 ± 5.00	89.22 ± 8.67	114.20 ± 6.05	84.56 ± 4.30
FE	102.59 ± 9.53 ^b^	99.76 ± 8.79	96.49 ± 11.01	115.65 ± 6.85	83.67 ± 4.29
FOL	97.52 ± 6.62 ^ab^	100.53 ± 11.16	93.10 ± 10.02	116.44 ± 6.51	84.28 ± 6.97
FEFOL	93.17 ± 9.56 ^ab^	102.64 ± 6.52	87.95 ± 10.82	112.32 ± 7.31	80.58 ± 6.00
10 days					
C	94.83 ± 5.49 ^ab^	83.64 ± 6.05	89.48 ± 6.56	112.22 ± 6.89 ^ab^	83.64 ± 6.05
D	91.48 ± 8.03 ^a^	78.37 ± 3.82	94.92 ± 12.80	110.98 ± 2.77 ^ab^	78.34 ± 3.82
FE	105.19 ± 7.24 ^b^	81.17 ± 3.99	86.42 ± 9.23	110.18 ± 6.82 ^ab^	81.18 ± 4.00
FOL	98.05 ± 10.66 ^ab^	81.05 ± 4.82	90.32 ± 9.34	113.34 ± 8.10 ^b^	81.05 ± 4.82
FEFOL	94.66 ± 8.31 ^ab^	80.58 ± 8.84	98.21 ± 9.93	104.09 ± 6.22 ^a^	80.58 ± 8.84
21 days					
C	87.36 ± 10.64	92.55 ± 13.64	92.36 ± 13.92 ^ab^	109.70 ± 7.90	81.62 ± 3.98
D	86.57 ± 12.07	97.29 ± 5.50	96.46 ± 8.77 ^ab^	111.04 ± 10.25	79.34 ± 3.71
FE	98.47 ± 12.45	97.69 ± 7.23	82.01 ± 7.06 ^a^	113.94 ± 9.81	82.98 ± 12.21
FOL	96.02 ± 7.94	93.78 ± 6.81	88.74 ± 12.80 ^ab^	113.20 ± 6.70	84.44 ± 3.69
FEFOL	98.16 ± 6.86	94.51 ± 3.78	99.60 ± 9.89 ^b^	105.52 ± 5.38	76.92 ± 5.66

*C* control diet, *D* diet deficient in iron and folic acid, *FE* diet deficient in folic acid and supplemented with iron, *FOL* diet deficient in iron and supplemented with folic acid, *FEFOL* diet supplemented with iron and folic acid, *d.w*. dry weight

^a, b, c^Significant differences (*p* < 0.05) between groups (within the columns); ANOVA and Tukey’s test

**Table 5:** Concentration of calcium (μg/g d.w.) in tissues in rats

Group	Liver	Spleen	Pancreas	Kidney	Heart
2 days					
C	41.43 ± 5.32 ^ab^	93.08 ± 17.92	134.55 ± 29.97 ^ab^	227.77 ± 59.78 ^b^	126.02 ± 28.68 ^b^
D	31.14 ± 4.46 ^a^	73.44 ± 13.64	125.23 ± 49.81 ^ab^	175.50 ± 21.00 ^ab^	86.56 ± 6.75 ^a^
FE	48.11 ± 8.67 ^b^	97.31 ± 30.03	129.62 ± 40.77 ^ab^	195.85 ± 35.09 ^b^	93.76 ± 9.89 ^ab^
FOL	45.77 ± 11.63 ^b^	76.15 ± 8.83	158.68 ± 22.10 ^b^	196.98 ± 29.05 ^b^	79.29 ± 11.98 ^a^
FEFOL	40.64 ± 16.38 ^ab^	71.45 ± 8.18	109.68 ± 42.47 ^a^	133.54 ± 39.35 ^a^	145.84 ± 39.79 ^b^
10 days					
C	28.66 ± 4.64 ^ab^	61.76 ± 23.67 ^ab^	127.43 ± 22.10 ^ab^	193.98 ± 33.66 ^b^	90.91 ± 13.17 ^bc^
D	20.99 ± 7.54 ^a^	47.03 ± 5.82 ^a^	122.25 ± 39.71 ^ab^	171.20 ± 33.68 ^ab^	65.48 ± 13.15 ^b^
FE	35.51 ± 5.91 ^ab^	105.93 ± 4.01 ^b^	93.18 ± 8.46 ^a^	133.44 ± 20.44 ^a^	41.21 ± 5.89 ^a^
FOL	43.07 ± 12.78 ^b^	99.94 ± 28.10 ^ab^	143.68 ± 17.02 ^ab^	195.61 ± 32.31 ^b^	80.54 ± 17.48 ^b^
FEFOL	41.42 ± 9.89 ^b^	99.72 ± 13.98 ^ab^	170.37 ± 55.77 ^b^	139.46 ± 26.34 ^a^	108.60 ± 14.86 ^c^
21 days					
C	42.56 ± 9.26 ^b^	109.73 ± 27.18 ^b^	165.83 ± 40.21	121.30 ± 45.57	111.25 ± 17.75 ^ab^
D	23.90 ± 4.12 ^a^	76.60 ± 6.56 ^a^	155.54 ± 26.53	156.35 ± 34.36	82.15 ± 10.21 ^a^
FE	66.33 ± 14.79 ^b^	103.05 ± 18.75 ^b^	121.64 ± 48.89	182.14 ± 26.93	103.99 ± 22.90 ^ab^
FOL	56.42 ± 6.72 ^b^	79.65 ± 8.01 ^a^	159.36 ± 20.67	223.75 ± 53.52	110.05 ± 18.25 ^b^
FEFOL	85.92 ± 13.33 ^c^	150.46 ± 19.98 ^c^	180.33 ± 46.46	197.09 ± 38.74	157.61 ± 17.66 ^c^

*C* control diet, *D* diet deficient in iron and folic acid, *FE* diet deficient in folic acid and supplemented with iron, *FOL* diet deficient in iron and supplemented with folic acid, *FEFOL* diet supplemented with iron and folic acid, *d.w*. dry weight

^a, b, c^Significant differences (*p* < 0.05) between groups (within the columns); ANOVA and Tukey’s test

**Table 6:** Concentration of magnesium (μg/g d.w.) in tissues in rats

Group	Liver	Spleen	Pancreas	Kidney	Heart
2 days					
C	1306.35 ± 63.62 ^b^	912.71 ± 40.86 ^a^	918.52 ± 62.22	804.66 ± 44.23 ^a^	950.30 ± 43.02
D	1247.14 ± 45.28 ^b^	928.64 ± 33.82 ^a^	910.93 ± 54.36	747.65 ± 20.65 ^a^	936.21 ± 25.48
FE	736.64 ± 34.43 ^a^	912.92 ± 30.44 ^a^	1009.86 ± 70.19	861.47 ± 24.66 ^b^	955.59 ± 41.04
FOL	717.65 ± 16.03 ^a^	929.67 ± 76.94 ^a^	1020.99 ± 175.08	848.53 ± 17.20 ^b^	1007.13 ± 79.12
FEFOL	682.20 ± 61.10 ^a^	1044.68 ± 61.94 ^b^	864.49 ± 136.48	893.44 ± 52.99 ^b^	1033.79 ± 188.78
10 days					
C	1291.99 ± 26.75 ^b^	905.87 ± 28.06 ^a^	943.77 ± 36.47	779.18 ± 35.14 ^a^	936.84 ± 21.15
D	1238.41 ± 53.93 ^b^	902.07 ± 41.23 ^a^	915.46 ± 54.14	756.15 ± 20.56 ^a^	905.41 ± 34.33
FE	753.63 ± 34.86 ^a^	892.91 ± 27.51 ^a^	937.86 ± 69.71	873.39 ± 28.39 ^b^	934.19 ± 27.67
FOL	704.28 ± 35.79 ^a^	859.26 ± 74.23 ^a^	945.65 ± 69.23	843.59 ± 12.87 ^b^	895.31 ± 26.90
FEFOL	712.21 ± 30.63 ^a^	1037.60 ± 30.42 ^b^	933.99 ± 91.72	834.43 ± 25.07 ^b^	989.42 ± 48.03
21 days					
C	1287.45 ± 89.48 ^b^	886.89 ± 123.38 ^ab^	880.13 ± 95.99	773.27 ± 38.70 ^a^	948.19 ± 24.03 ^ab^
D	1242.90 ± 154.09 ^b^	894.97 ± 51.57 ^ab^	909.54 ± 70.09	742.06 ± 34.27 ^a^	932.91 ± 28.32 ^a^
FE	719.55 ± 30.96 ^a^	870.43 ± 46.98 ^a^	938.06 ± 73.84	845.56 ± 29.89 ^b^	933.80 ± 48.42 ^a^
FOL	720.32 ± 25.80 ^a^	854.75 ± 58.16 ^a^	928.49 ± 65.39	867.40 ± 20.06 ^b^	966.57 ± 27.43 ^ab^
FEFOL	729.87 ± 28.50 ^a^	975.67 ± 49.09 ^b^	935.11 ± 47.67	841.61 ± 23.64 ^b^	987.91 ± 40.94 ^b^

*C* control diet, *D* diet deficient in iron and folic acid, *FE* diet deficient in folic acid and supplemented with iron, *FOL* diet deficient in iron and supplemented with folic acid, *FEFOL* diet supplemented with iron and folic acid, *d.w*. dry weight

^a, b, c^Significant differences (*p* < 0.05) between groups (within the columns); ANOVA and Tukey’s test

After 2 days of supplementation, significant changes in copper occurred only in the heart, while the lowest level was found in the FEFOL group and the highest in the control group (Table 3). The intermediate period of supplementation (10 days) showed more changes in copper level in tissues: a significantly lower level of copper was noted in the liver and heart in the FOL group; however, the pancreases and kidneys of the FE group showed a markedly lower concentration of this element than the control group. Long-term supplementation caused a significant decrease in copper in the liver, pancreas, kidney, and heart in the Fe group, in comparison to the control group. Markedly lower concentrations were also found in the heart in FOL group than in the C group. We showed that a deficit of iron and folate did not markedly change the concentration of copper in the rat tissues (group D). Ten and 21 days of simultaneous iron and folic acid supplementation did not markedly affect copper level in the organs. Separate supplementation with iron and folic acid significantly reduced the copper levels in most tissues we analyzed, compared to the control group (Table 3).

After 2 and 10 days of intervention, we found the highest level of zinc in the FE group and the lowest in the D group, in the liver (Table 4). A significantly higher level of zinc was found in the FOL group than in the FEFOL group in the kidney, in the second stage of the intervention. After 21 days of supplementation, the only marked change was seen in the pancreas, where the FE group had lower concentrations that the FEFOL group. In the case of zinc, it was found that neither the deficiency of iron and folic acid nor their supplementation altered the content of this element in the tissues in relation to the C group.

The calcium content of tissues is shown in Table 5. As can be seen, the concentration depends on the tissue, group, and stage. After 2 days of supplementation, the lowest level of calcium was seen in the D group in the liver, in the FEFOL group in pancreas and kidney, and in the FOL group in the heart; the highest level of calcium was found in the FE group in the liver, in the FOL group in the pancreas, in the control group in the kidney, and in the FEFOL group in the heart. After 10 days of supplementation, the lowest concentration of calcium was found in the D group in the liver and spleen and in the FE group in the pancreas, kidney, and heart; the highest value was noted in the FOL group in the liver and kidney and in the FEFOL group in the pancreas and heart; the spleen had the highest value of calcium content in the FE group. The long-term iron and folic acid deficit led to significantly decreased calcium levels in the liver and spleen, and simultaneous iron-folic acid supplementation markedly increased calcium content in the FEFOL group in the liver, spleen, and heart, compared to the control group.

The concentration of magnesium in tissues is shown in Table 6. After 2 days of supplementation, the level of Mg in the liver and kidney was significantly lower in the supplemented groups than in groups D and C. In the spleen, a markedly higher level of Mg was found in the FEFOL group. This trend continued in in the liver, kidney, and spleen in the subsequent stages. After 21 days of the intervention, markedly lower levels of Mg were observed in the D and FE groups than in the FEFOL group.

Correlation analysis between the level of iron and of other elements in the tissues was performed in each stage, and for the whole period of study (Table 7). The short intervention time of 2 days showed a significant negative correlation between the content of iron and zinc in the spleen (*r* = − 0.33) and kidney (*r* = − 0.37), and also between iron and magnesium in the spleen (*r* = − 0.34). A positive correlation was found between iron and calcium concentration in the spleen (*r* = 0.54). After 10 days of the intervention, a positive correlation was seen between iron concentration and copper in the heart (*r* = 0.60) and zinc in the liver (*r* = 0.48). Long-term supplementation showed a negative relationship between the content of iron and copper in the kidney (*r* = − 0.55) and magnesium in the liver (*r* = − 0.40), and a positive correlation was found in the liver for zinc (*r* = 0.52) and calcium (*r* = 0.61). The entire period of the study showed a marked relationship between iron and the other elements at all stages, and a negative correlation was seen between iron and copper in the spleen (*r* = − 0.41) and kidney (*r* = − 0.55), and magnesium in the liver (*r* = − 0.40). A positive correlation was seen between calcium and iron in the liver (*r* = 0.42).

**Table 7:** Significant correlation (*r*) between iron* and minerals content (μg/g d.w.) in tissues

Element	Iron				
	Liver	Spleen	Pancreas	Kidney	Heart
2 days					
Copper	NS	NS	NS	NS	NS
Zinc	NS	− 0.33	NS	− 0.37	NS
Calcium	NS	0.54	NS	NS	NS
Magnesium	NS	− 0.34	NS	NS	− 0.36
10 days					
Copper	NS	NS	NS	NS	0.60
Zinc	0.48	NS	NS	NS	NS
Calcium	NS	NS	NS	NS	NS
Magnesium	NS	NS	NS	NS	NS
21 days					
Copper	NS	NS	NS	− 0.36	NS
Zinc	0.52	NS	NS	NS	NS
Calcium	0.61	0.49	NS	NS	NS
Magnesium	− 0.40	Ns	NS	NS	NS
Whole period					
Copper	NS	− 0.41	NS	− 0.55	NS
Zinc	NS	NS	NS	NS	NS
Calcium	0.42	NS	NS	NS	NS
Magnesium	− 0.40	NS	NS	NS	NS

^*^Iron concentration in tissues was shown in our previous paper [6]; *d.w.* dry weight; *NS* non-significant; Pearson correlation coefficient (*r*), significant difference at *p* < 0.05

## Discussion

In this study, we found that separate and simultaneous supplementation of iron and folate affects tissue copper, zinc, calcium, and magnesium concentration in rats after short-, moderate-, and long-term intervention. To the best of our knowledge, this is the first study to present the interaction between iron–folate supplements and minerals.

The results of this study show that a deficit in iron and folic acid decreases calcium concentration in the liver, spleen, and heart. The longer intervention revealed copper reduction in the liver and heart in the deficient group. Moreover, we observed that iron and folic acid supplementation decreased the copper and magnesium levels, but increased the calcium level, in some tissues of rats deficient in iron and folic acid. However, the effect on copper and calcium status depended on the type of supplementation: simultaneous supplementation affected calcium, while iron and folic acid taken separately affected copper.

The changes seen in this study may indicate interactions between minerals. This can be confirmed by the significant correlation between iron and other elements found in the tissues.

The only variable factors in this study were dietary iron and folic acid level. The levels of other minerals were identical in the diets. The concentration of iron in tissues has been described in our previous paper [15], which found that folic acid may affect iron status in moderate and long-term supplementation with these micronutrients. This interaction between folic acid and iron might affect other minerals, and thus our results.

It is known that the interaction between elements may have significant effects on iron status and on the outcome of iron supplementation in an Fe-deficient state.

In a human study conducted by Tiwari et al. [16], a significant increase in copper and a slight increase in zinc levels were observed after iron and folic acid supplementation. Moreover, the authors also found that iron deficiency anemia led to a decrease in the essential trace minerals and that iron and folic acid supplementation recovered these metals. Our study partly confirms these results, as we found significantly lower levels of copper in the liver and heart after 10 and 21 days of intervention. It was also shown that simultaneous iron and folic acid supplementation recovered copper status, but that iron and folic acid separately usually decreased copper in the tissues. This different effect of iron and folic acid on copper—depending on whether they were used separately or together—is rather unexpected. It may be associated with the interaction between iron and folic acid. The level of iron in the diet was low in the deficient group (D group) and also in the folate group (FOL group), and when iron and folic acid are used simultaneously, the effect is to decrease the iron content of tissues, as we showed in our previous work [15]. In that study, we also observed copper ion shifts between the liver, kidney, and heart with moderate- and long-term interventions, and this may also have affected our results. This could reflect an opposite interaction between iron and copper in the spleen and kidney, and a positive effect in the heart. The interaction we observed between iron and copper partly confirmed the results of our previous experimental study, where copper levels decreased in the serum and tissues of the rats fed the high-iron diet; this was associated with a disturbance of carbohydrate metabolism [17]. Other studies also showed that iron excess was associated with lower copper levels in serum and tissues, and this was connected with ceruloplasmin and iron metabolism [18, 19].

In this study, we unexpectedly found no effect of iron content in the diet (whether deficit or excess) on zinc status. However, we did find a positive correlation between iron and zinc content in the liver in the moderate-term and long-term interventions. Taking into account the results of the other studies, we assumed that our interventions had a negative influence on zinc status. It has been observed in some human studies that iron supplementation may decrease zinc uptake and zinc concentration in serum [12, 20]. It has also been suggested that folic acid, particularly in high doses, may reduce zinc absorption and impair zinc utilization [21]. Shankar et al. [20] found that weekly iron and folic acid supplementation led to the development of hypozincemia in both anemic and nonanemic pregnant women. This study did not confirm that result, which may have been caused by the duration of the intervention or an insufficient iron dose, or may also be associated with the interaction between iron and folic acid. It this study, we additionally observed the shift of zinc ions between tissues, especially from the liver to the pancreas, along with the time of intervention; this could also have affected our results.

We observed large fluctuation in the calcium content of organs, depending on the stage of the experiment. A markedly positive correlation between the iron and calcium content of tissues was established in the final stage of the experiment. The interaction between calcium and iron was mainly associated with the absorption stage and with the ion shift between tissues and redistribution in the body [22, 23]. Negative effects of the interaction between iron and calcium were usually observed, especially when one of the elements was taken in excess. The effect of iron overload on calcium malabsorption has been observed in thalassemia [24]. It was found that iron hyperabsorption leads to impaired calcium transport through deregulation of calciotropic hormone production and response, low transcellular calcium uptake, aberrant hepcidin release and response, and overexpression of DMT1 or ferroportin-1. This interaction may also include regulators of calcium absorption, such as FGF-23 [24]. A link has also been shown between low calcium concentration and anemia [25]. The results of this study may have been affected by the combination of folic acid with iron in the supplement, and also by the low dose of iron and the use of separate or simultaneous supplementations in iron-folic acid deficient rats.

The interactions between magnesium and iron alone, and combined with folic acid, have been rather poorly described in human and animal studies. Our study has shown that the deficit of iron and folic acid did not affect magnesium status, but that each kind of supplementation (micronutrients alone or taken together) significantly reduced the concentration of magnesium in tissues. Shankar et al. [20] reported that weekly iron and folic acid supplementation decreased magnesium serum concentration in women. It seems that these results are associated with the iron content of the organ, because we found a negative correlation between magnesium and iron in the liver in the last stage and over the whole experimental period.

In our discussion of mechanisms affecting mineral status here, we should mention that phytate lowers the availability of Fe, Zn, and other minerals. Added Fe-gluconate may supersede the phytate content from the diet, release Ca and Mg from the phytate, and prevent the formation of Zn-phytate (which may be more stable than Fe-phytate) [26].

Our study has some limitations: we examined only one dose of iron and folate in the supplements and only one form of iron (iron gluconate). These data did not include other folic acid, iron, or mineral status parameters. We did not assess the mechanism of interaction between iron, folic acid, and the elements.

In conclusion, iron–folic acid supplementation, whether separate or simultaneous, affects copper, calcium, and magnesium levels of tissues. The status and supply of minerals should therefore be considered when iron–folic acid supplements are used for prevention or therapy. Our results need further exploration in humans.
